# Spectroscopy, Structure, Biomacromolecular Interactions, and Antiproliferation Activity of a Fe(II) Complex With DPA-Bpy as Pentadentate Ligand

**DOI:** 10.3389/fchem.2022.888693

**Published:** 2022-04-25

**Authors:** Hehe Bai, Jia Shi, Qingyu Guo, Wenming Wang, Zhigang Zhang, Yafeng Li, Manohar Vennampalli, Xuan Zhao, Hongfei Wang

**Affiliations:** ^1^ Key Laboratory of Chemical Biology and Molecular Engineering of the Education Ministry, Institute of Molecular Science, Shanxi University, Taiyuan, China; ^2^ The Fifth Hospital (Shanxi Provincial People’s Hospital) of Shanxi Medical University, Taiyuan, China; ^3^ Department of Chemistry, University of Memphis, Memphis, TN, United States

**Keywords:** Fe complex, structure, biomacromolecules, cytotoxicity, pentadentate ligand

## Abstract

An Fe(II) complex with DPA-Bpy (DPA-Bpy = N,N-bis(2-pyridinylmethyl)-2,20-bipyridine-6 -methanamine) as the ligand was synthesized and characterized to mimic bleomycin. The binding constants (*K*_b_) of the complex with calf thymus DNA and human serum albumin (HSA) were quantitatively evaluated using fluorescence spectroscopy, with *K*_b_ as 5.53×10^5^ and 2.40×10^4^ M^−1^, respectively; the number of the average binding site (*n*) is close to 1. The thermodynamic analyses suggested that the electrostatic interactions exist between the complex and DNA, and the hydrogen bonding and Van der Waals force exist for the complex and HSA. The Fe complex exhibits cleavage ability toward pBR322 DNA, and the crystal structure of the HSA Fe complex adduct at 2.4 Å resolution clearly shows that His288 serves as the axial ligand of the Fe center complexed with a pentadentate DPA-Bpy ligand. Furthermore, the cytotoxicity of the complex was evaluated against HeLa cells. Both the Fe complex and HSA Fe complex adduct show obvious effect on cell proliferation with an IC_50_ of 1.18 and 0.82 μM, respectively; they induced cell apoptosis and arrested cell cycles at S phase. This study provides insight into the plausible mechanism underlying their metabolism and pharmacological activity.

## Introduction

Platinum-based drugs have been successfully used in the treatment of various types of cancer in clinic (Breglio et al., 2017; Shaili et al., 2019; Karmakar et al., 2020; Tham et al., 2020; Wan et al., 2021). However, these agents exhibit undesired side effects during chemotherapy. Therefore, non-platinum–based complexes of Ru, Os, Fe, etc., become alternative candidates for treating cancer, and a number of contributions on the synthesis and anticancer activity of these metal complexes were reported (Chen et al., 2017; Monro et al., 2019; Icsel et al., 2020; Odachowski et al., 2020; Loginova et al., 2020; Bolitho et al., 2021; Wang et al., 2021). Iron (Fe) is an essential bio-metal element in life systems and plays key roles in metabolism and biochemical reactions (Andrews, 1999). Previous studies have shown that rapidly growing tumor cells, such as leukemia and neuroblastoma, have particularly high demands for Fe (Iancu et al., 1988; Le and Richardson, 2002; Gou et al., 2016a). Therefore, it is important to investigate the potent anticancer activity of Fe complexes and elucidate their mechanism of action in order to discover novel leading compounds.

**SCHEME 1:**
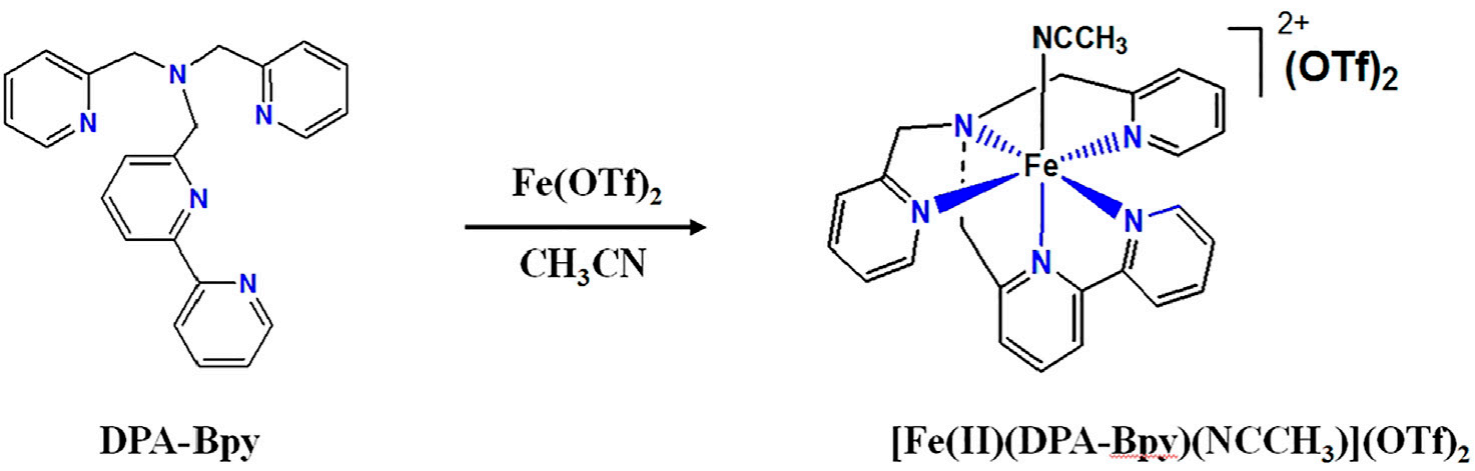
Synthetic route and structure of [Fe(II)(DPA-Bpy)(NCCH_3_)](OTf)_2_.

Moreover, both the type of organic ligands and nature of coordinated metal ions seem to be responsible for their pharmacological activities. The pentadentate ligands are appropriate and ideal candidates for mimics of bleomycin, which is a well-known antitumor drug. Bleomycin is a classical metallo-glycopeptide antibiotic that contains ligands with five nitrogen donors; it requires a transition metal ion, usually Fe(II), for activity; and the cytotoxicity of bleomycin is believed to be related to DNA breakage, and Fe(II)-bleomycin shows the highest DNA cleavage activity (Ma et al., 2009; Myers et al., 2013; Murray et al., 2018). In this work, an Fe complex with a pentadentate ligand, N,N-bis(2-pyridinylmethyl)-2,20-bipyridine-6-methanamine (DPA-Bpy), was prepared. Scheme 1 shows the synthetic routes and structure of the [Fe(II)(DPA-Bpy)(NCCH_3_)](OTf)_2_ complex. The physicochemical characteristics, antiproliferative effect, and possible interactions with bio-macromolecules were investigated.

Although the accepted targets for the Fe bleomycin complex and platinum antitumor drugs are nuclear DNA, increasing evidence indicates that proteins also play important roles in the display of antitumor activity (Rao et al., 1980; Chen and Stubbe, 2005; Pinato et al., 2014; Moghaddam et al., 2015; Messori and Merlino, 2016; Morais et al., 2016; Zou et al., 2017; Geersing et al., 2018; Cheng et al., 2019; Sacco et al., 2021; Sakharkar et al., 2021). Human serum albumin (HSA) is the most abundant protein in blood, and it serves as a transporter molecule for a variety of endogenous compounds, including metal drugs. Thus, the binding of complexes with HSA have a significant impact on their cytotoxicity and provides a model for understanding the interactions with proteins. Hereby, cytotoxicity of the Fe complex was assessed *in vitro* on human cervical cancer cells (HeLa cells). Moreover, the interactions between the complex and bio-macromolecules, including DNA and HSA, were analyzed using spectroscopic and structural methods. The analyses for their interactions can provide evidence on a plausible mechanism underlying their metabolism and pharmacological activity.

## Experimental Section

### Reagents and Syntheses

Calf thymus DNA (CT-DNA), pBR322 DNA, ethidium bromide (EB) solution, and albumin human serum (HSA) were purchased from Solarbio (Beijing, China). DMEM medium, fetal bovine serum, penicillin/streptomycin, and PBS buffer solution used in cell culture were purchased from Sangon Biotech (Shanghai, China). The Cell Counting Kit-8 (CCK-8) was purchased from Dojindo (Kumamoto, Japan). Human cervical cancer cells (HeLa cells) were provided from Shanxi Key Laboratory of Pharmaceutical Biotechnology (Taiyuan, China). Other chemicals and solvents used were purchased from locally available suppliers.

Synthesis of the [Fe(II)(DPA-Bpy)(NCCH_3_)](OTf)_2_ complex: The synthesis of the pentadendate ligand, N,N-bis(2-pyridinylmethyl)-2,20-bipyridine-6-methanamine (DPA-Bpy) was achieved by our previous method (Radaram et al., 2011; Singh et al., 2012). The [Fe(DPA-Bpy)(NCCH_3_)](OTf)_2_ complex was prepared with a modified method. The reaction of Fe(OTf)_2_ (145 mg, 0.40 mmol) with DPA-Bpy (150 mg, 0.40 mmol) in CH_3_CN (30 ml) at room temperature for overnight results in a reddish cloudy solution. The precipitate was filtrated and washed with Et_2_O and then dried under vacuum to yield [Fe(DPA-Bpy)(NCCH_3_)](OTf)_2_ as a dark red powder (Scheme 1). Yield: 270 mg, 89%. ^1^H NMR (CD_3_CN): 9.10 (d, 1H, *J* = 5.4 Hz, bipy-H13), 8.57 (d, 1H, *J* = 8.0 Hz, bipy-H10), 8.32 (d, 1H, *J* = 7.9 Hz bipy-H9), 8.29 (td, 1H, *J* = 8.0, 1.4 Hz bipy-H11), 7.87 (t, 1H, *J* = 8.0 Hz, bipyH-12), 7.82 (td, 1H, *J* = 7.9, 1.4 Hz, bipy-H8), 7.71 (td, 2H, *J* = 7.9 1.4 Hz, pyr-H3), 7.42 (d, 2H, *J* = 7.9 Hz, pyr-H2), 7.26 (d, 1H, *J* = 7.9 Hz, bipy-H7), 7.0 (t, 2H, *J* = 6.6 Hz, pyr-H4), 6.83 (d, 2H, *J* = 5.6 Hz, pyr-H5), 4.95 (d, 2H, *J* = 15.9 Hz, pyrCH_2_), 4.86 (d, 2H, *J* = 15.9 Hz, pyrCH_2_), 4.91 (dd, 4H, *J* = 15.9 Hz, pyrCH_2_), and 4.75 (s, 2H, bipyCH_2_). ESI-MS: *m/z* ^+^ 572.30 (Calcd *m/z* ^+^ for [Fe(DPA-Bpy)(OTf)]^+^, 572.07). Anal. Calcd for C_27_H_24_F_6_FeN_6_O_6_S_2_: C, 42.53; H, 3.17; N, 11.02. Found: C, 42.40; H, 3.09; N, 10.79. NMR and MS spectra for the complex are submitted as Supplementary Material.

### Measurements

^1^H NMR spectra were measured by the Bruker 500 MHz NMR spectrometer. Elemental analysis of the [Fe(DPA-Bpy)(NCCH_3_)](OTf)_2_ complex was conducted by Atlantic Microlab, Inc., Atlanta, Georgia. The mass spectra were obtained by the Thermo Q Exactive field and Bruker Ultraflex MALDI-TOF MS spectrometer. UV–Vis spectra were recorded on a Thermo Evolution-220 spectrometer. Fluorescence spectra were recorded on a FluoroMax-4 spectrofluorometer.

### Binding of the Fe Complex With DNA

Fluorescence spectroscopy was measured to analyze the interaction of the Fe complex with CT-DNA. The concentration of the CT-DNA stock solution was calculated by determining the absorption at 260 nm. Then, the CT DNA-EB solution was prepared by mixing 2.5 μM EB and 170 μM CT-DNA in a 10 mM Tris buffer (pH 7.4). Finally, the titrations were conducted by the addition of the Fe complex (0–20 μM) to the CT-DNA-EB solution step by step until a plateau in the fluorescence intensity was reached. The sample solution was stirred gently at room temperature, and the fluorescence spectra were collected in the range of 530–750 nm using 1-cm quartz every 5 min. The excitation wavelength was 520 nm, and slit width was set to 5 nm.

The interaction of the Fe complex with plasmid DNA was analyzed by agarose gel electrophoresis. The supercoiled pBR322 DNA alone was used as the control and then pBR322 DNA (2 μg) and the Fe complex (10–50 μM) were mixed and the buffer solution was added to a total volume of 20 μl. The mixed solutions were protected from light and incubated for 30 min. Finally, the solutions were electrophoresed at 70 V through 1% agarose gel immersed in 1× TBE buffer for 40 min.

### Binding of the Fe Complex With HSA Protein

The binding of the Fe complex with HSA was explored by the fluorescence quenching method and MALDI-TOF MS spectra. Different concentrations of the complex (0–50 μM) were added to HSA (10 μM) in 20 mM PB buffer (pH 7.4), and then the fluorescence spectra were recorded in a range of 290–520 nm with 280 nm as the excitation wavelength. Both the slit-width for excitation and emission were set to 5 nm. 2,5-dihydroxybenzoic acid (DHB) was used as the matrix for MALDI-TOF MS spectral measurement. HSA (5 mg/ml) and the Fe complex (the ratio was 1:5) were mixed in 20 mM PB buffer (pH 7.4) and kept at 4°C in the refrigerator overnight. The mass spectra were recorded from 30,000 to 150,000.

### Structural Determination of HSA and the Fe Complex Adduct

Crystallization of HSA was performed with the hanging-drop vapor diffusion method at 18°C. The drops consisted of 1.5 μl of the HSA solution and 1.5 μl of reservoir solution. The crystallization reservoir contains 25% (w/v) polyethylene glycol 3,350, 5% glycerol, 5% DMSO, and 50 mM potassium phosphate (pH 7.5). The native crystals were soaked in a solution containing 5 mM Fe complex, 25% (w/v) PEG 3350, and 25 mM potassium phosphate buffer for 5 h. The X-ray diffraction dataset to a maximum resolution of 2.4 Å was collected at the Shanghai Synchrotron Radiation Facility (SSRF, Shanghai, China). The data were processed, merged, and scaled with the HKL-3000 (Minor et al., 2010). The structure was determined by molecular replacement using the Phaser program in the Phenix program package (McCoy et al., 2007). The coordinates of HSA and [RuCl_5_(indazol)]^2-^ complex adduct (PDB ID: 5GIY) were used as an initial model (Zhang et al., 2014). The structure was rebuilt using COOT (Emsley and Cowtan, 2004), and the structural refinement was conducted using the program Phenix (Adams et al., 2002). The model geometry was verified using MolProbity (Chen et al., 2010), and the structural figures were drawn using PyMOL (DeLano, 2002).

### Cytotoxic Activity

The cytotoxicity of the Fe complex was evaluated against HeLa cells using CCK-8 assay. HeLa cells were cultured with a DMEM adding 10% (v/v) FBS and 1% (v/v) penicillin/streptomycin at 37°C under 5% CO_2_ atmosphere. The cells were seeded on a 96-well plate with a density of 5 × 10^4^ cells/ml. After they were incubated for 12 h, the cultured cells were treated with the culture medium containing different concentrations of the Fe complex (0, 0.5, 1, 2, 5, and 10 μM) and incubated for another 24 h. Then, 100 μl CCK-8 solution (1 mg/ml) diluted with the cell culture medium was added to each well, and the plate was further incubated for 3 h. The absorbance was recorded at 450 nm using a SpectraMax iD5 microplate reader. Each experiment was repeated at least three times. The IC_50_ values of the complex against the HeLa cell were calculated by plotting the viability versus concentration on a logarithmic scale.

The effects of the complex on the HeLa cell cycle and apoptosis were analyzed by the flow cytometric technique. Cell cycle arrest was performed by staining the cell with PI. Apoptosis assays were evaluated using Annexin V-FITC as an apoptosis detection kit.

## Result and Discussion

### Synthesis and Characterization

The Fe complex structure was confirmed from different spectroscopy techniques including ^1^H–NMR, elemental analysis, ESI–MS, and UV–Vis as described in the experimental section and supplementary information (Supplementary Figures S1–S3). At room temperature, the ^1^H–NMR spectrum in acetonitrile indicates a diamagnetic Fe complex. The ^1^H–NMR spectrum of the complex (Supplementary Figure S1) shows the two pyridine groups with the same NMR features, suggesting the presence of *C*_s_ symmetry in a solution with a plane containing the tertiary amine and bipyridine group. The DPA-Bpy is a strong-field ligand; it stabilizes and forms the low spin iron (II) complex [Fe(II)(DPA-Bpy)(NCCH_3_)](OTf)_2_. The Fe complex is highly soluble in water, while DPA-Bpy ligand is insoluble.

The mass spectrometry appearance of the molecular ion at 572 provided further evidence for the metal complex in positive mode corresponding to [Fe(DPA-Bpy)(OTf)]^+^ (Supplementary Figure S2). The central Fe atom has six-coordinated structures with one DPA-Bpy ligand and one solvated ligand, similar to that of [Ru(DPA-Bpy)Cl]Cl and [Co(DPA-Bpy)Cl]Cl (Radaram et al., 2011; Singh et al., 2012). As shown in Supplementary Figure S3, the UV–Vis spectrum of the complex in acetonitrile shows two obvious absorption bands at 350–600 nm from charge transfer transitions between the metal ion and ligand.

### DNA Binding and Breakage Properties

Since the potential and vital biological targets of metal complexes are DNA and proteins, the interactions of metal complexes with DNA/protein have attracted great interest in determining the mode of binding. The binding analyses for metal complexes and DNA is the key to understanding the mechanism of their pharmacological action (Murray et al., 2018; Shao et al., 2021). Interactions of the Fe complex with CT-DNA were studied by fluorescence spectroscopy and agarose gel electrophoresis.

**FIGURE 1:**
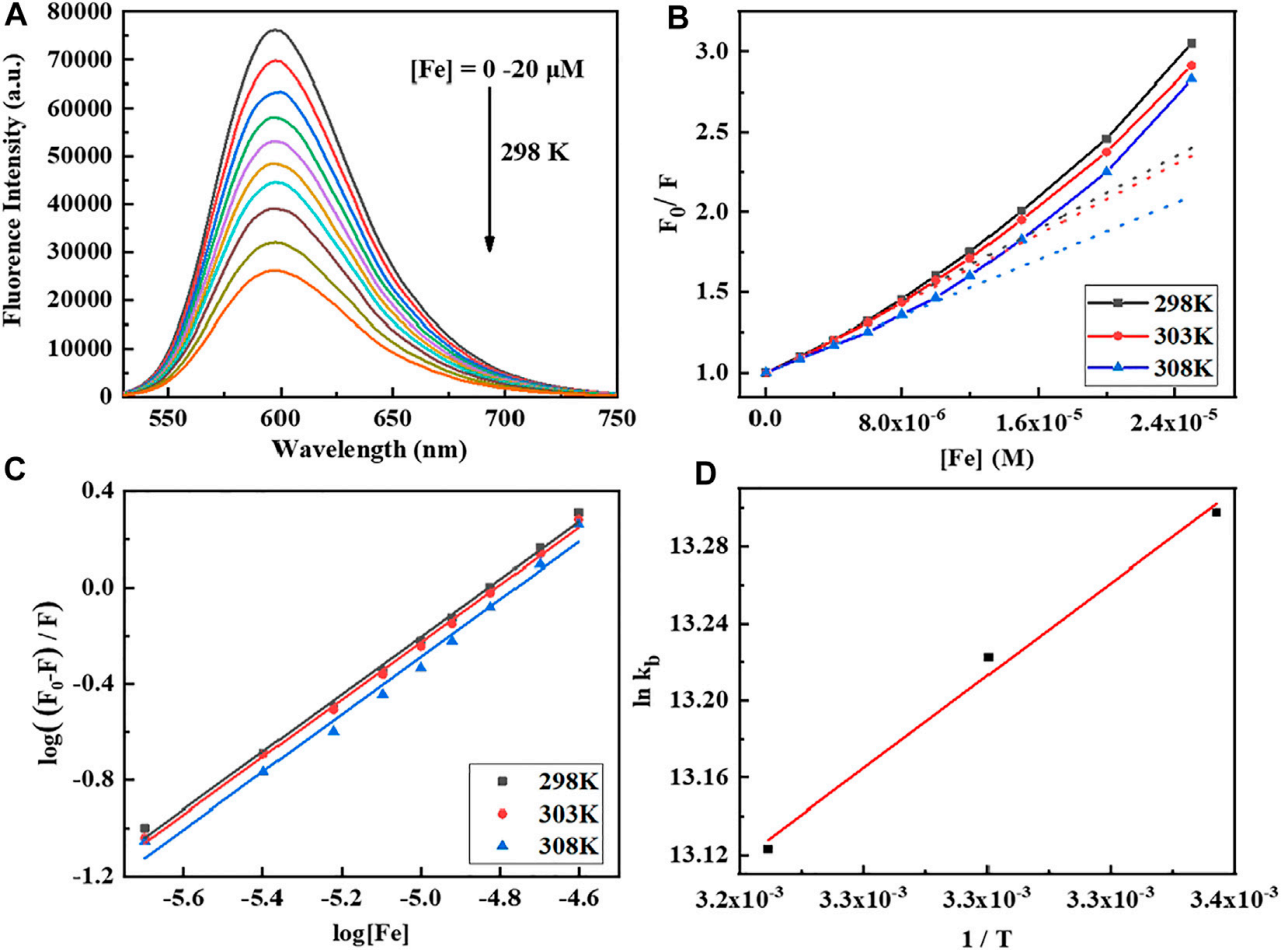
**(A)** Fluorescence emission spectra of CT-DNA (170 μM) titration with the Fe complex by increasing concentrations (0–20 μM). The excitation wavelength is 520 nm at pH 7.4 and 298 K. **(B,C)** Stern–Volmer plot of CT-DNA quenched by the Fe complex at different temperatures. **(D)** Plot of ln*K*_b_ vs. 1/T for the interaction of the Fe complex with CT-DNA.

Fluorescence spectroscopy is a sensitive technique to analyze the binding pattern and strength of the complex with CT-DNA using EB as a fluorescence probe. The strong emission intensity at 598 nm for the CT-DNA-EB adduct was observed when the EB aromatic ring is inserted into the base pair of DNA double helix. However, the fluorescence intensity decreases obviously after the addition of the Fe complex, and due to that, the complex replaces EB in the EB-DNA system (Figure 1). The binding constant (*K*_b_) and the number of average binding sites (n) are calculated according to the Stern–Volmer Eqs 1, 2 (Seyedi et al., 2020; Shao et al., 2021).

$$\frac{F_{0}}{F}=1+K_{Q}\tau_{0}\left[Q\right]=1+K_{SV}\left[Q\right].$$ (1)

$$\text{log}\left({\text{F}}_{\text{0}}-\text{F}\right)\text{/F }=\text{ log }K\text{b}+\text{n log}\left[\text{Q}\right]\;\text{.}$$ (2)

To better understand the thermodynamics of the reaction between the Fe complex and DNA, the changes of standard entropy (∆S), enthalpy (∆H), and Gibbs free energy (∆G) are further evaluated at three different temperatures according to Eq. 3. The results are listed in Table 1. The *K*_b_ is 5.00–5.96 × 10^5^ M^−1^, and the average binding-site number is about 1.2. The positive value of ∆S (and ∆H near to 0) suggested that electrostatic interactions exist between the complex and CT-DNA (Yang et al., 2011; Heydari and Mansouri-Torshizi, 2016; Seyedi et al., 2020; Shao et al., 2021). The negative value of ∆G shows the spontaneity of the reaction, and the negative value of ∆H reveals that the interaction of the Fe complex with DNA is exothermic. This is in agreement with decrease in the *K*_b_ values upon increasing the temperature (Table 1).

ΔG=ΔH - ΔS = -RT ln Kb . (3)

**TABLE 1:** Binding constant (*K*_b_) and number of average binding sites (*n*) of the Fe complex with DNA at different temperatures.

T (K)	*K*_sv_ (M^−1^ s^−1^)	*R* ^2^	*K*_b_ (M^−1^)	*n*	*R* ^2^	∆H (kJ/mol)	∆S (J/mol)	∆G (kJ/mol)
298	5.649×10^12^	0.996	5.957×10^5^	1.196	0.996	−12.299	65.967	−32.957
303	5.445×10^12^	0.997	5.527×10^5^	1.194	0.998			−33.287
308	4.405×10^12^	0.997	5.004×10^5^	1.197	0.998			−33.616

**FIGURE 2:**
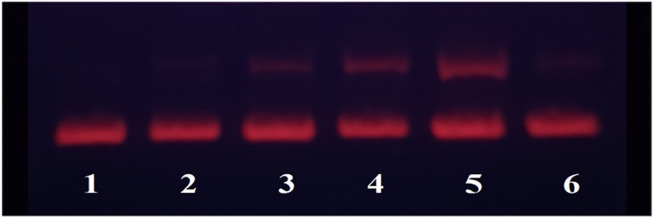
Agarose gel electrophoresis of pBR322DNA (0.1 μg) with different concentrations of the Fe complex. Lane 1: DNA only; Lane 2: 0.1 μM Fe-DPA-Bpy; Lane 3: 1.0 μM Fe-DPA-Bpy; Lane 4: 10.0 μM Fe-DPA-Bpy; Lane 5: 50.0 μM Fe-DPA-Bpy; and Lane 6: 10 μM DPA-Bpy.

In order to investigate the effect of the Fe complex on the configuration of DNA, the technique of agarose gel electrophoresis was used, and the results are shown in Figure 2. The plasmid pBR322 DNA with different configurations moves at different velocities in the electric field. The closed superhelical form (CC) migrates faster in the electrophoresis process, while the open-circular form (OC) moves slower because of the single-strand breakage (Zhou et al., 2009). There is just the CC form for DNA alone in Lane 1 for control. As the concentration of the Fe complex increased, more OC forms of DNA were observed in Lanes 2–4. The 10 mM Fe complex led to an obvious interaction of DNA, while almost no damage was observed for the ligand alone at the same concentration. It indicated that the coordination of Fe with the DPA-Bpy ligand is necessary to induce DNA cleavage, in which it is possible through a free radical mechanism such as bleomycin Fe complex (Ma et al., 2009; Myers et al., 2013; Murray et al., 2018).

### Protein Binding and the Thermodynamic Properties

Human serum albumin (HSA) is the most abundant protein in plasma and is the main carrier of endogenous and exogenous ligands, including fatty acids (FAs), nucleic acids, hormones, metals, toxins, and drugs. Thus, HSA plays a relevant role as a drug carrier by influencing their pharmacokinetics and pharmacodynamics (Ishima et al., 2012; Belinskaia et al., 2021; De Simone et al., 2021). Furthermore, the fluorescence spectroscopy and MALDI-TOF MS spectra were used to study the binding mode and interaction between the synthesized complex and HSA.

**FIGURE 3:**
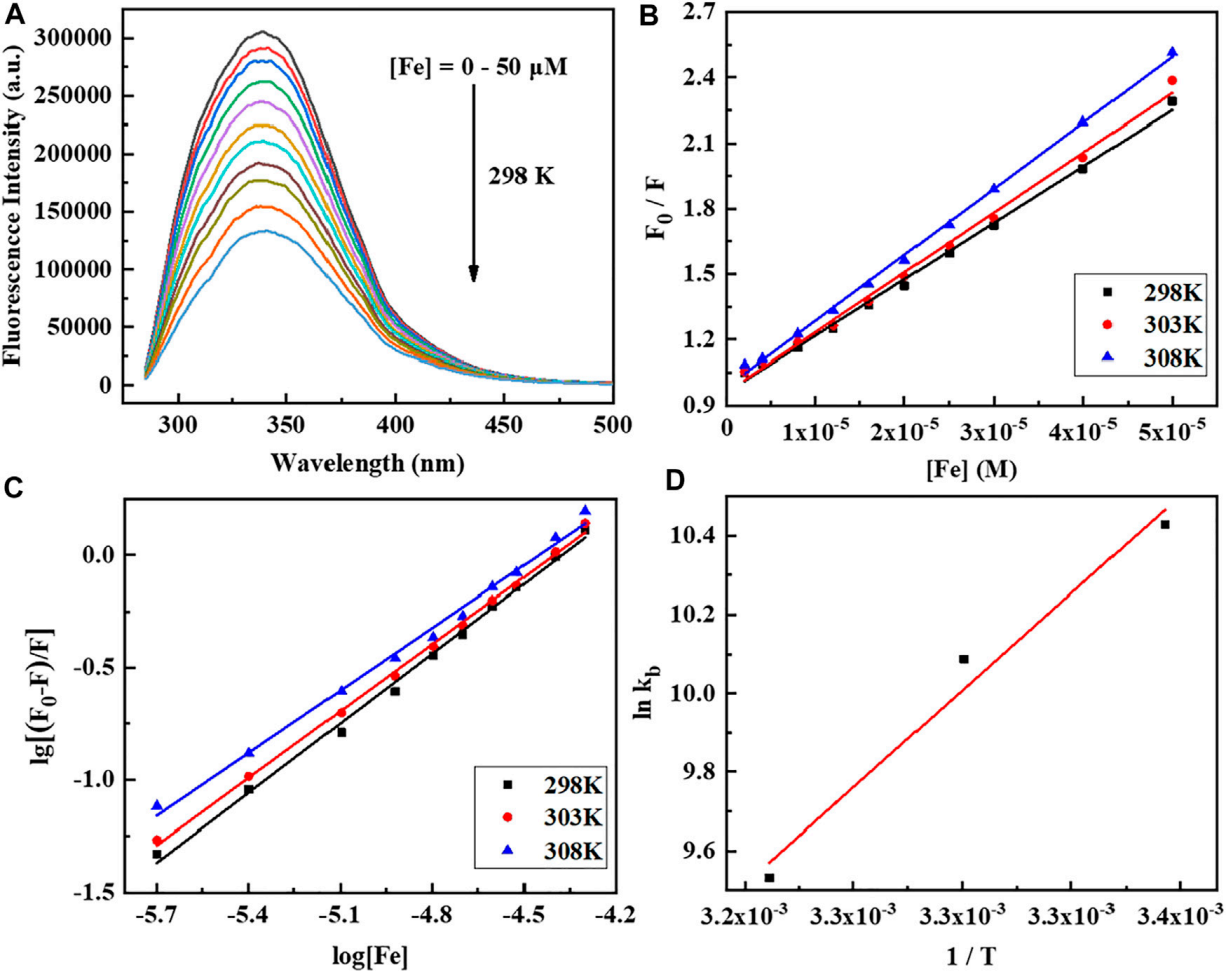
**(A)** Fluorescence emission spectra of human serum albumin (HSA) (10 μM) titration with the Fe complex by increasing concentrations (0–50 μM). The excitation wavelength is 280 nm at pH 7.5 and 303 K. **(B,C)** Stern–Volmer plot of HSA quenched by the Fe complex at different temperatures. **(D)** Van’t Hoff plot of ln*K*_b_ vs. 1/T for the interaction of the Fe complex with HSA.

The HSA is intrinsically fluorescence active which comes from Trp, Tyr, and Phe residues. As shown in Figure 3, the fluorescence is very sensitive to its microenvironment, where the fluorescence quenching of HSA is observed with increasing concentrations of the Fe complex. It indicated that the molecular interactions and binding of the Fe complex with serum protein affects the chromogenic groups.

The Stern–Volmer Eq. 2 is used to analyze the fluorescence quenching data at three temperatures, and the corresponding calculated results are listed in Table 2. The *K*_b_ is 1.34–3.09 × 10^4^ M^−1^, and the average binding-site number is close to 1. The calculated ∆H, ∆S, and ∆G are shown in Table 2. The negative values of ∆S and ∆H are frequently taken as evidence for the hydrogen bond and van der Waals power. The negative value of ∆G shows the spontaneity of the reaction. The increase in the *K*_b_ values upon increasing the temperature and the positive value of ∆H reveals that the binding process of the Fe complex with HSA is endothermic (Zhou et al., 2009; Yang et al., 2011; Shao et al., 2021).

**TABLE 2:** Binding constant (*K*_*b*_) and number of average binding sites (n) of the Fe complex with HSA.

T (K)	*K*_sv_ (×10^12^, M^−1^ s^−1^)	*R* ^2^	*K*_b_ (×10^5^, M^−1^)	*n*	*R* ^2^	∆H (kJ/mol)	∆S (J/mol)	∆G (kJ/mol)
298	2.580	0.996	3.379	1.035	0.996	−68.321	−142.25	−25.929
303	2.736	0.995	2.404	0.995	0.998			−25.218
308	3.018	0.998	1.378	0.930	0.994			−24.507

**FIGURE 4:**
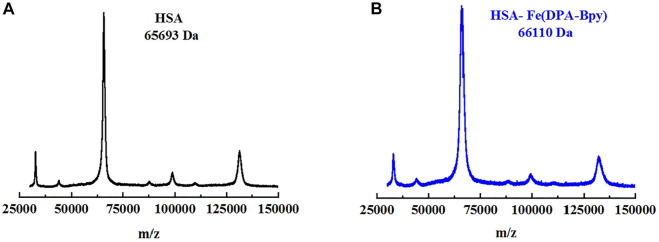
MALDI-TOF MS spectra of HSA and HSA Fe complex adduct in DHB matrix. **(A)** HSA alone (black line) and **(B)** HSA Fe complex adduct (blue line).

The HSA molecule is a single-chain protein which contains 585 amino acid residues in a heart-like shape, and the three domains in HSA provide binding sites for a wide variety of endogenous ligands (Ghuman et al., 2005; Xie et al., 2021). To examine the number of the Fe complex bound to HSA in solution, the native and Fe complex–treated HSA were analyzed by MALDI-TOF MS. The MS spectra for native HSA and Fe-HSA complex adduct are shown in Figure 4; the spectra in the DHB matrix revealed that the m/z of native HSA was 65,694 and that of the HSA incubated with the Fe complex was 66,110. The difference of the m/z value is 416, which is just close to the molecular fragment mass (423.3) of one [Fe (DPA-Bpy)] complex.

### Crystal Structure of HSA and Fe Complex Adduct

HSA plays a key role in regulating the distribution of many natural and artificial small molecules. The interaction of the complex with HSA may provide information for understanding their biodistribution, metabolic process, and pharmacological mechanisms of complexes (Zhu et al., 2008; Yamaguchi et al., 2010; Wang et al., 2013; Qi et al., 2016; Ito et al., 2020). To further investigate the binding mode of the Fe complex to HSA, the crystal structure of HSA and the Fe complex adduct was determined by using the X-ray diffraction method. The structure was refined to 2.4 Å resolution, and the coordinate has been deposited to the Protein Data Bank (PDB ID: 7WLF). The final refinement statistics are summarized in Table 3.

**TABLE 3:** Crystallographic data collection and model refinement statistics for HSA Fe complex adduct.

Parameters	
PDB code	7WLF
a (Å)	179.50
b (Å)	38.16
c (Å)	95.78
α, β, γ (°)	90, 105.08, 90
Space group	*C 1 2 1*
Wavelength used(Å)	0.9789
Resolution(Å)	36.99–2.40
No. of all reflections	453,848
No. of unique reflections	24,561
Completeness (%)	98.3 (98.3)
Average I/σ (I)	21.71 (1.93)
R_merge_ ^a^ (%)	7.5
No. of reflections used (σ(F) > 0)	24,780 (2,399)
R_work_ ^b^ (%)	22.1
R_free_ ^b^ (%)	27.2
R.m.s.d. bond distance(Å)	0.010
R.m.s.d. bond angle°	1.29
Average B-factor (Å^2^)	75.0
No. of protein atoms	4,562
No. of solvent atoms	94
Ramachandran plot	
Res. in favored regions (%)	95.5
Res. in generously allowed region (%)	4.5
Res. in disallowed region (%)	0

a R_merge_ = Σ_h_Σ_l_ | I_ih_−<I_h_> |/Σ_h_Σ_I_ <I_h_>, where < I_h_> is the mean of the observations I_ih_ of reflection h.

b R_work_ = Σ(||F_p_(obs)|−|F_p_(calc)||)/Σ|F_p_(obs)|; R_free_ is an R factor for a preselected subset (5%) of reflections that was not included in refinement. ^c^ Numbers in parentheses are corresponding values for the highest resolution shell.

**FIGURE 5:**
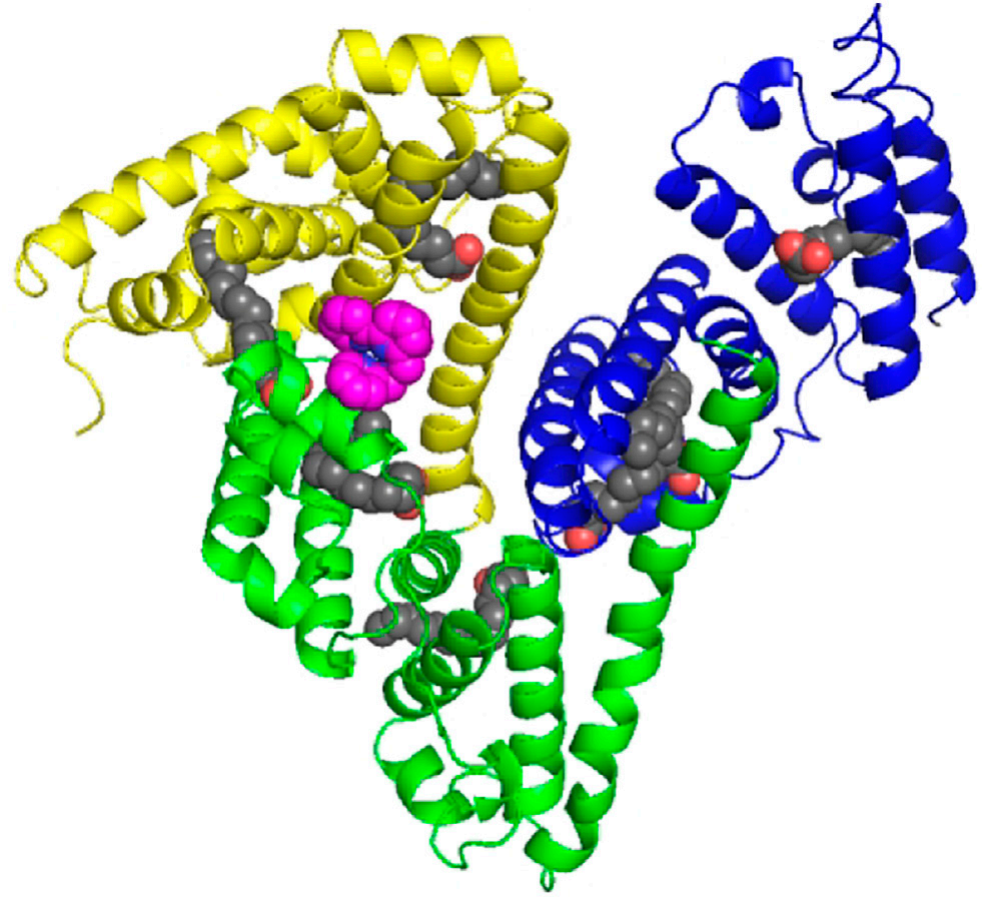
Cartoon representation of the overall structure of HSA and Fe complex adduct. Every domain is differently colored (domain I, yellow; domain II, green; and domain III, blue). The Fe complexes are represented as spheres in pink. The seven palmitic acid (PA) molecules bound to HSA are displayed as sphere chains (aliphatic chain, gray spheres; carboxylate oxygens, red spheres).

**FIGURE 6:**
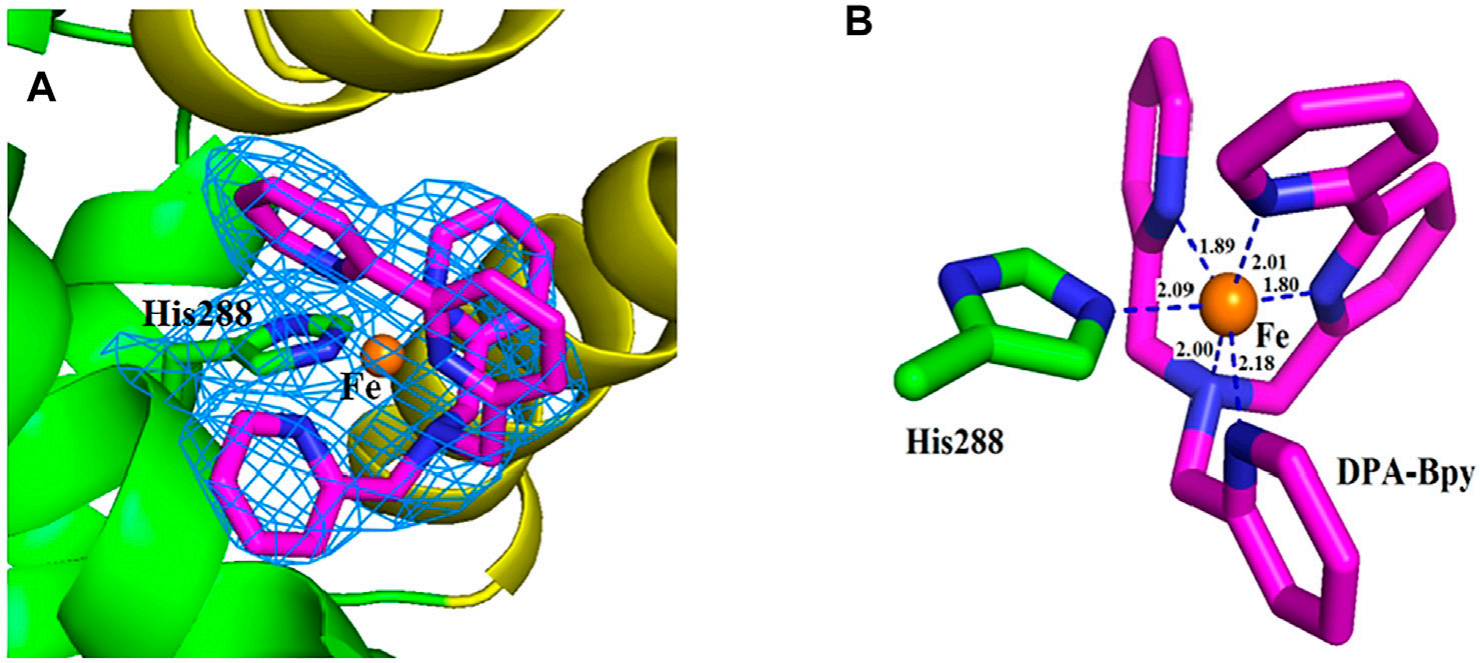
Close view of the electron density map (2*Fo−Fc*) of the binding site for the Fe complex. The map is contoured at 1.0 σ and shown as a skyblue mesh **(A)**. Nearest residue to the Fe complex is shown as green sticks. The ligand molecule is shown as pink sticks and Fe ion is shown as brown spheres **(B)**. Distances between the atoms are shown as blue dash lines; respectively.

The overall structure is shown in Figure 5, and the crystal structure indicates that HSA has three typical domains (I–III). The refined electron density map clearly revealed that one Fe complex molecule is coordinated with His288 and binding in site between the interface of domain I and domain II. The Fe center is coordinated with 1 N atom of His288 and 5 N atoms of DPA-bpy ligand. The six bond lengths between Fe and N atoms are in the range 1.80–2.18 Å, as shown in Figure 6. The binding site is located near the center region of the heart structure of HSA and exposed to the surface of the protein. This site is just a little above the well-known warfarin binding sites (called as drug binding site 1 in domain II, PDB: 2BXD) of HSA (Ghuman et al., 2005; Zhu et al., 2008).

The Fe complex is located at the pocket delimited by residues including Glu153, Phe156, Phe157, Lys159, Arg160, Lys281, Glu285, and Glu292. The occupancy of the metal center is 0.46, and the B-factors for the Fe atom is 64.87. The binding site is different with the reported HSA complex structures of other Ru complexes, Cu(II) and Fe(III) complexes. There are two binding sites for the [RuCl_5_(ind)]^2-^ complex (Zhang et al., 2014); the Ru atoms coordinated with His146 in the IB subdomain and His242 and Lys199 residues in the IIA subdomain of HSA. For a Cu(II) complex with tridentate (E)-N'-(5-bromo-2-hydroxybenzylidene) benzohydrazide Schiff base ligand (Gou et al., 2016b) and Fe(III) complex containing the tridentate 2-hydroxy-1-naphthaldehyde thiosemicarbazone Schiff base ligand (Qi et al., 2016), the central metal ions were coordinated with His242 and Lys199 residues of HSA by replacing the other two ligands of the complexes. In addition, seven palmitic acid (PA) molecules are present in the subdomains of I–III and in the border region between domains II and III.

### Cytotoxicity of the Fe Complex

**FIGURE 7:**
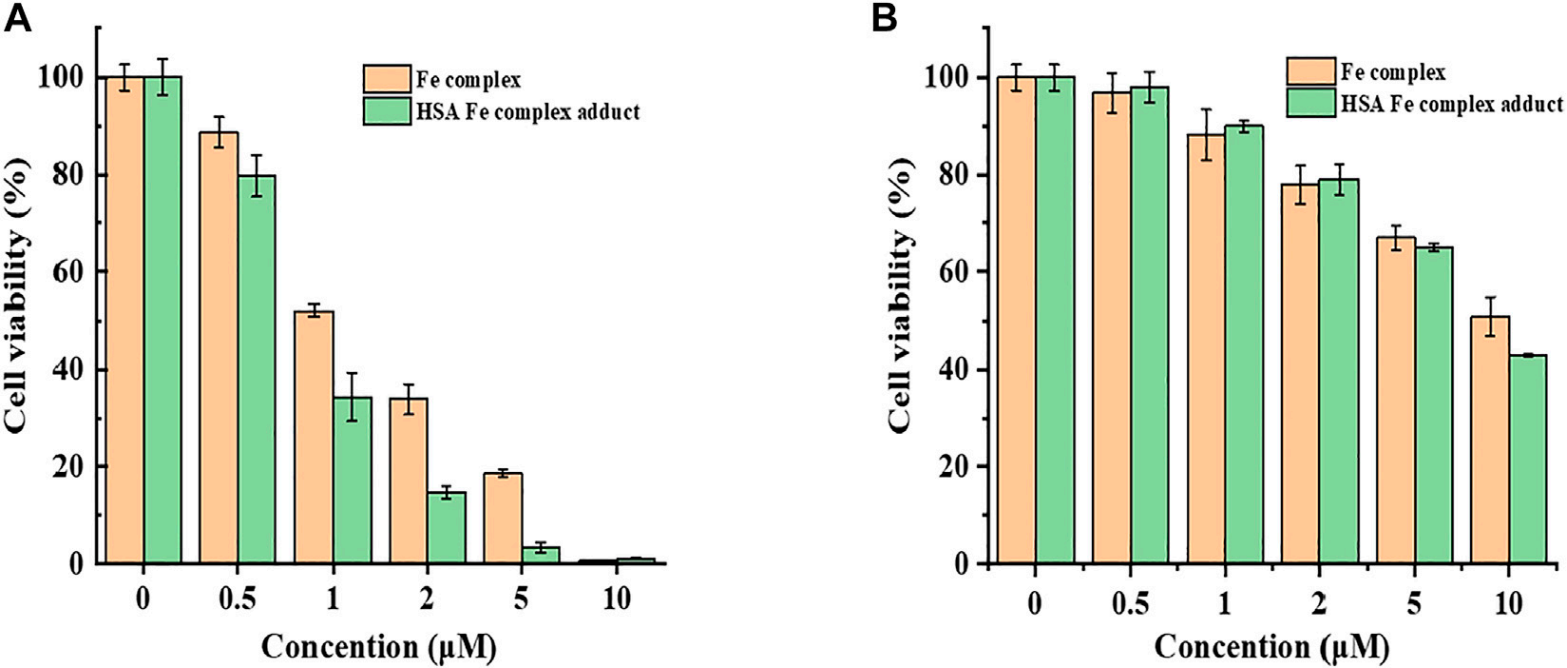
Cell viability of the Fe complex and HSA Fe complex adduct on HepG2 cells with CCK-8 **(A)**. Comparison of the cytotoxicity of the Fe complex with that of HSA Fe complex adduct on cell HL7702 **(B)**.

Antitumor and antiproliferative activities of the first series of transition metal complexes containing different ligands have been reported (Chen et al., 2017; Monro et al., 2019; Icsel et al., 2020; Loginova et al., 2020; Odachowski et al., 2020). The iron as a common, abundant, and important element has attracted great attention for its cytotoxicity and genotoxicity. Though the mechanism of action for Fe complexes is complicated, studies on the interactions of metal complexes with bio-macromolecules possibly shed light on the mechanism of activity and metabolism. Cytotoxicity of the Fe complex and Fe complex HSA adduct was evaluated against HeLa cells using the CCK-8 method. Figure 7 showed the effect of the Fe complex and its HSA complex adduct on the cell viability of HeLa cells and normal liver cell HL-7702, where the mortality of HeLa cells increased as the concentrations of the Fe complex and its HSA complex adduct increased. The complex had a certain inhibitory effect on the growth of the cancer cells, and the IC_50_ of the Fe complex and its HSA complex adduct was 1.18 and 0.82 μM, respectively, for the tested tumor cell, while the Fe complex and its HSA complex adduct had a less inhibitory effect on the normal liver cell HL-7702 at similar concentration ranges.

**FIGURE 8:**
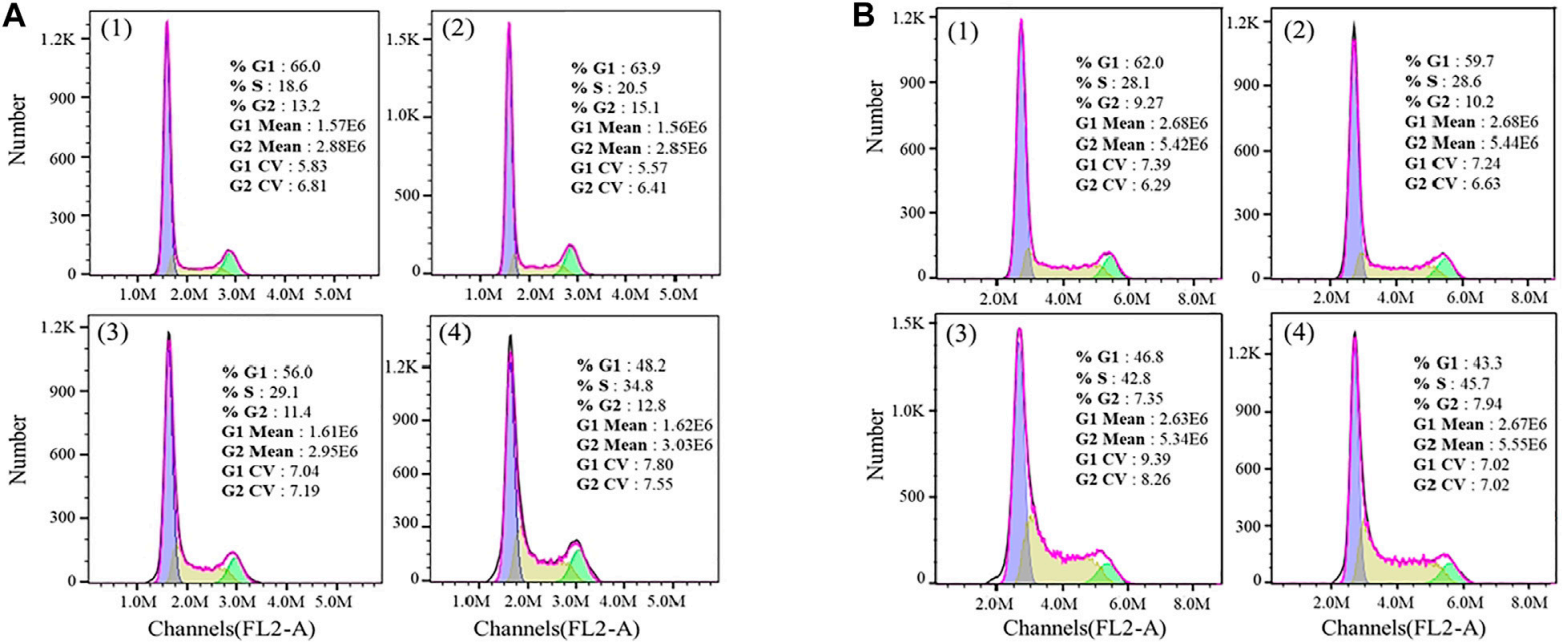
Effects of the Fe complex **(A)** and the HSA Fe complex adduct **(B)** on the cell cycles. (1: 0 μM; 2: 0.2 μM; 3: 0.5 μM; and 4: 0.8 μM).

**FIGURE 9:**
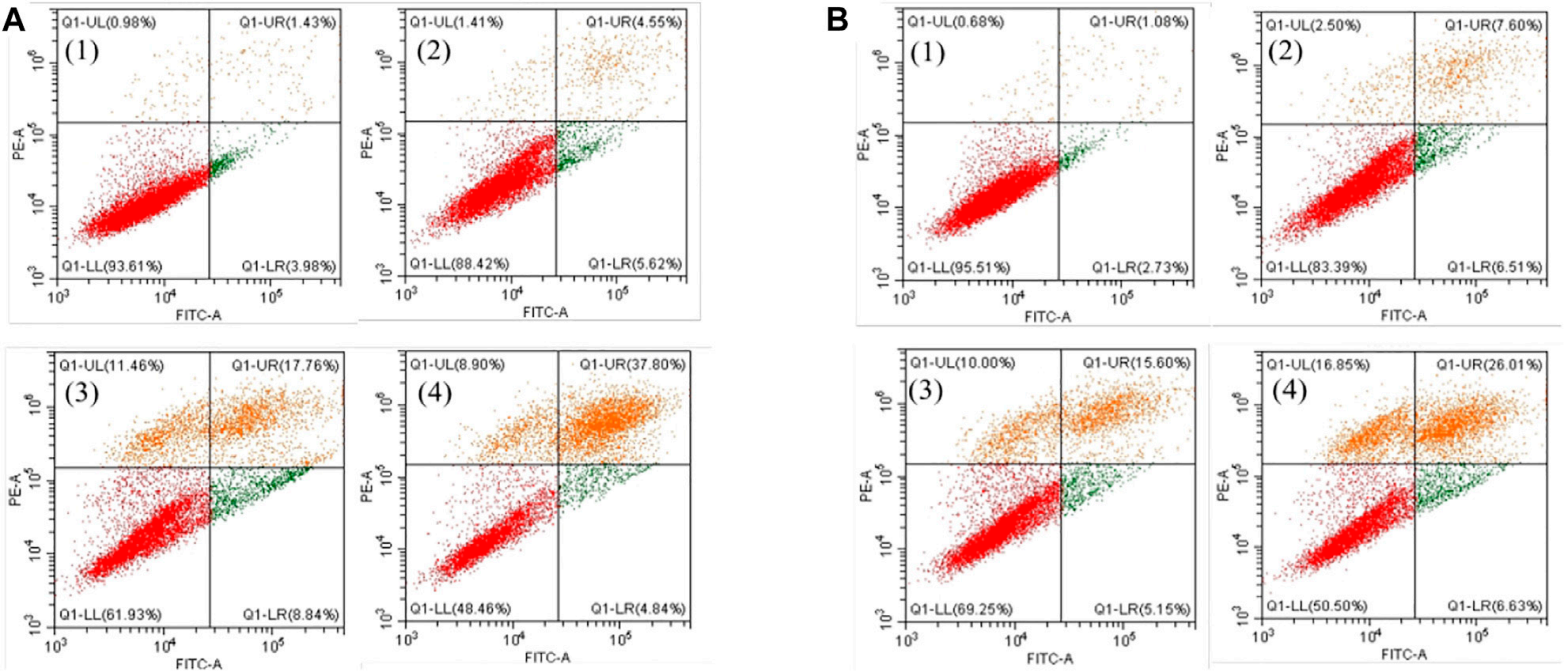
Effects of the Fe complex **(A)** and the HSA Fe complex adduct **(B)** on the apoptosis of HeLa cells. (1: 0 μM; 2: 0.5 μM; 3: 0.8 μM; and 4: 1.0 μM).

Furthermore, the flow cytometric data were analyzed to examine the effects of the Fe complex on HeLa cell cycle distribution. As shown in Figure 8A, the percentage of G1 phase for the negative control is 66.0%, and it decreases to 56.0 and 48.2% at concentrations of 0.5 and 0.8 µM, respectively. The percentages of cells in the S phase increased from 18.6 to 29.1% and 34.8% at the same concentrations. These results indicated that the Fe complex induced a dose-dependent S phase arrest of HeLa cells. It is similar to that of the Fe complex with heterocyclic thiosemicarbazone ligands (Gou et al., 2016a). For the Fe complex HSA adduct (Figure 8B), the percentage of G1 phase decreases from 62.0 to 46.8% and 43.3% at concentrations of 0.5 and 0.8 µM, respectively. The percentages of cells in the S phase increased from 28.1 to 42.8% and 45.7% at the similar concentrations. The effects of the Fe complex and the HSA Fe complex adduct on the apoptosis of HeLa cells were shown in Figure 9. The Fe complex HSA adduct showed a higher inhibitory effect than that of the Fe complex alone, which is consistent with their cytotoxicity.

## Conclusion

In summary, an Fe complex with DPA-Bpy as the ligand was synthesized, and it shows obvious cytotoxicity on proliferation of HeLa cells. The IC_50_ of the Fe complex and its HSA complex adduct is 1.18 and 0.82 μM; respectively. The thermodynamic analyses suggested that electrostatic interactions exist between the complex and DNA, and the hydrogen bonding and van der Waals force exist for the complex and HSA. Moreover, the Fe complex exhibits cleavage ability toward pBR322 DNA; and the crystal structure of the HSA Fe complex adduct clearly shows that one pentadentate DPA-Bpy coordinated Fe center is bound by His288 as the axial ligand. HSA could be a delivery carrier for the complex. Both the Fe complex and HSA Fe complex adduct induced cell apoptosis and arrested cell cycle at the S phase. This study provided knowledge for the rational design of metal complex–based drugs and sheds light on the mechanism of action.

## Data Availability

The datasets presented in this study can be found in online repositories. The names of the repository/repositories and accession number(s) can be found in the article/Supplementary Material.
