# Supplementary material for: Spectroscopy, Structure, Biomacromolecular Interactions, and Antiproliferation Activity of a Fe(II) Complex With DPA-Bpy as Pentadentate Ligand
Source: Front Chem. 2022 Apr 25;10:888693. doi: 10.3389/fchem.2022.888693 (PMC9081768; doi:10.3389/fchem.2022.888693)
Supplement: Supplementary file 1 [file DataSheet1.docx]

**Supporting Information**

**Spectroscopy, Structure, Biomacromolecular Interactions and Anti-proliferation Activity of a Fe(II) Complex with DPA-Bpy as Pentadentate Ligand**

Hehe Bai ^a^, Jia Shi ^a^, Qingyu Guo ^a^, Wenming Wang ^a^, Zhigang Zhang ^a^, Yafeng Li ^b^,

Manohar Vennampalli ^c^, Xuan Zhao ^c^, Hongfei Wang ^a^

^a^ Key Laboratory of Chemical Biology and Molecular Engineering of the Education Ministry,

Institute of Molecular Science, Shanxi University, Taiyuan 030006, China

^b^ The Fifth Hospital (Shanxi Provincial People's Hospital) of Shanxi Medical University, Taiyuan,

030012, China

^c^ Department of Chemistry, University of Memphis, Memphis, TN 38152, USA


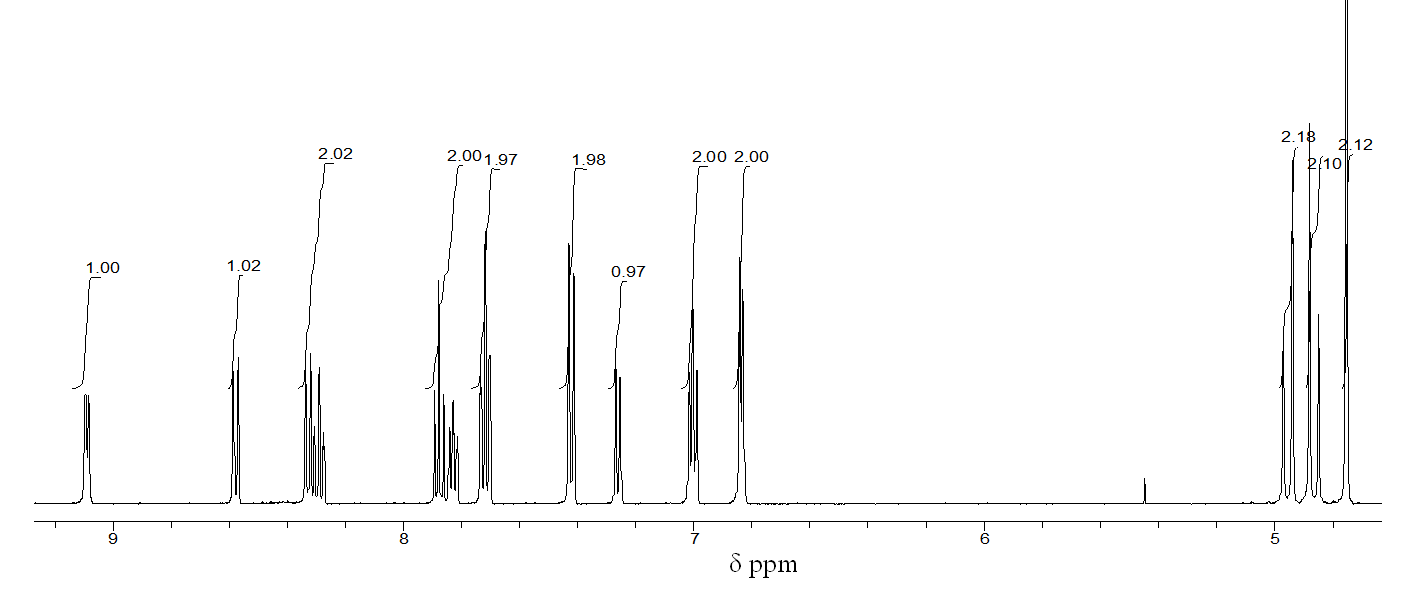


**FIGURE S1.** ^1^H NMR spectra (CD_3_CN) for [Fe(DPA-Bpy)(NCCH_3_)](OTf)_2_.


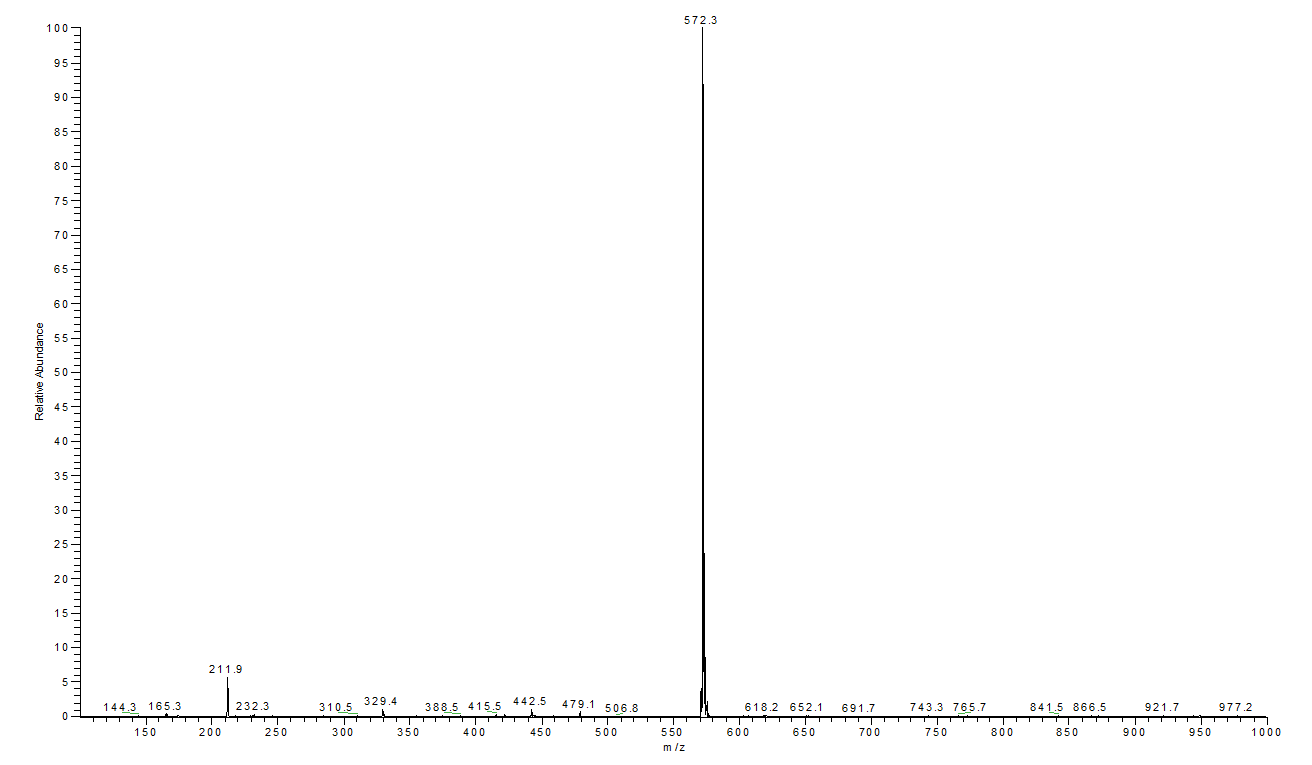


**FIGURE S2.** Mass spectrum of [Fe(DPA-Bpy)(NCCH_3_)](OTf)_2_ (**1**).

**
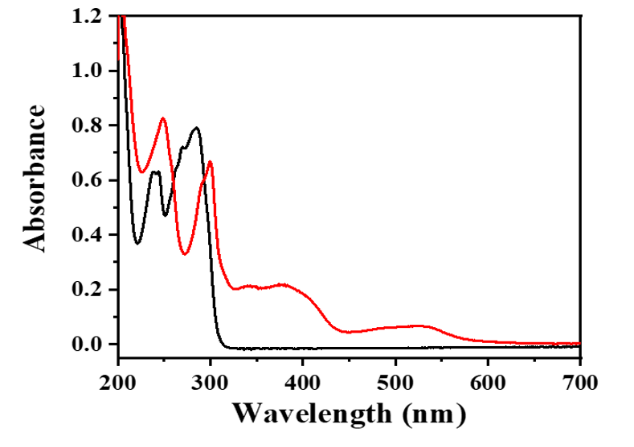
**

**FIGURE S3.** UV-Vis spectra of [Fe(DPA-Bpy)(NCCH_3_)](OTf)_2_ complex (red line) and DPA-Bpy ligand in CH_3_CN.
